# Development of full-body rhythmic synchronization in middle childhood

**DOI:** 10.1038/s41598-024-66438-7

**Published:** 2024-07-08

**Authors:** Jessica Phillips-Silver, Martin Hartmann, Laura Fernández-García, Nahuel Cruz Gioiosa Maurno, Petri Toiviainen, María Teresa Daza González

**Affiliations:** 1 JPS Research & Education, Washington, DC USA; 2 Centre of Excellence in Music, Mind, Body and Brain, University of Jyväskylä, Jyväskylä, Finland; 3 Department of Music, Art and Culture Studies, University of Jyväskylä, Jyväskylä, Finland; 4 Department of Psychology, University of Almería, Almería, Spain; 5 CIBIS Research Center, University of Almería, Almería, Spain

**Keywords:** Rhythmic entrainment, Rhythmic synchronization, Child development, Motion capture, Cognitive neuroscience, Human behaviour

## Abstract

Rhythmic entrainment is a fundamental aspect of musical behavior, but the skills required to accurately synchronize movement to the beat seem to develop over many years. Motion capture studies of corporeal synchronization have shown immature abilities to lock in to the beat in children before age 5, and reliable synchronization ability in adults without musical training; yet there is a lack of data on full-body synchronization skills between early childhood and adulthood. To document typical rhythmic synchronization during middle childhood, we used a wireless motion capture device to measure period- and phase-locking of full body movement to rhythm and metronome stimuli in 6 to 11 year-old children in comparison with adult data. Results show a gradual improvement with age; however children’s performance did not reach adult levels by age 12, suggesting that these skills continue to develop during adolescence. Our results suggest that in the absence of specific music training, full-body rhythmic entrainment skills improve gradually during middle childhood, and provide metrics for examining the continued maturation of these skills during adolescence.

## Introduction

Moving to music is an ancient and universal human behavior: across the world and throughout history we have moved to the beat together^1,2^. This phenomenon, referred to as rhythmic entrainment, at once encapsulates the tendencies to perceive and move in time with an external stimulus (such as an auditory beat or rhythm: temporal entrainment), to feel an emotional connection (affective entrainment), and to synchronize our rhythmic movements with another individual or a group (social entrainment)^3,4^. In more common parlance, it is easy to notice people everywhere, from infants to adolescents to the elderly, feeling the groove^5–7^. In fact, the extent to which the groove is felt in an individual can be predicted by their level of bodily (or sensorimotor) synchronization^8^. Thus, rhythmic synchronization is an important tool for understanding how the human brain—and body—are wired for music.

Rhythmic entrainment consists of a set of timing-based sensorimotor processes. In particular, moving to music can involve auditory, motor (planning & execution), proprioceptive (perception of body position), vestibular (movement against gravity, balance), visual and vibrotactile systems^9–15^. Typically, a combination of these systems will enable the brain to predict, and the body to execute, movement with precise, musically relevant timing. For example, a musician in a band will listen to other ensemble members and observe their visual cues in order to play in sync; the audience will feel the vibrations of the bass through speakers and floor which, in combination with their auditory perception of the sounds will cause them to clap, sway or dance along to the beat; and all who are moving in time together will not just hear the beat but will feel it in their bodies, as well as feeling the shared social and emotional experience of being “locked in'' to the music together. Even adults without special musical training are able to adjust their movements to a range of musical tempi, and can produce movements (traditionally measured by finger tapping) at varying metrical levels of the beat (i.e., at fractions or multiples of the musical pulse)^16–19^. Thus various, timing-based sensorimotor components of music give rise to the pervasive and varied human experience of rhythmic entrainment. As fundamental and universal as rhythmic entrainment seems to be, the scaffolding of these varied components may actually take quite a lot of time and experience to develop.

Sensitivity to rhythmic timing through sensorimotor experiences is present from early infancy, and seems to develop slowly with age and experience. Newborns detect a periodic beat structure in a sequence of tones^20,21^, young infants can discriminate between metrical beat patterns^22^, and they engage in turn-taking during interactions with their caregivers^23–25^. Music captivates young children’s attention, and they respond to its emotional content and social context^26–28^. Infants and toddlers spontaneously move their bodies in response to music, they can adjust their tempo (i.e., rate of movement) somewhat according to the stimulus, and their degree of rhythmic coordination is higher in the presence of displays of positive affect^29–32^. Not only do infants and children delight in moving their bodies to music (like rocking, bouncing, swinging, swaying and spinning), but those behaviors contribute to the development of the auditory, vestibular and motor systems together^33^. Because of the nature of those auditory-vestibulomotor interactions, by six months of age infants show the ability to “feel the beat” in music: that is, body movement on different beats of an ambiguous rhythm pattern causes them to recognize versions of that rhythm with acoustic accents corresponding to their movement^34^. At one year, the experience of having moved in synchrony to a rhythm with a stranger increases the toddlers’ prosocial behavior, as measured by their tendency to help the other^35^.

So we see evidence that children enjoy moving their bodies to music, and in doing so, they learn information about the music and about their relationships with others. But the extent to which individual children *successfully synchronize their full body movement with the beat of the music*—meaning that they detect the regular beat in the music and coordinate their motor action with it—is still largely unknown, especially after age 5. While some observational cases can be found of precocious synchronization ability in toddlers^36^, the available literature on empirical measures of corporeal rhythmic synchronization shows limited abilities in young children under 5 years of age. In one study, 2 and 4 year olds showed body movements (hopping, circling and swaying) that were sometimes periodic, only occasionally synchronized with the musical beat period, and did not show any adjustment for tempo^37^. In a study examining performance while drumming, young children showed minimal period- and phase-locking ability only beginning to emerge at age 4.5 years^26^, results which were consistent with data from traditional sensorimotor synchronization tapping studies in young children^38,39^.

After early childhood there is a near total lack of data available on full body rhythmic synchronization until adulthood, when studies show high levels of synchronization ability in adults^40^. What has been more widely studied is finger tapping as an index of sensorimotor synchronization ability. In tapping studies adults show high and stable levels of synchronization^41–43^, and children show an improvement in timing—tapping closer to the beat, thus reducing the asynchrony between sound and tap onset, and decreasing variability—with age^44–49^, and with music practice or training^50^. In sum, between early childhood and adulthood, some components of temporal entrainment and sensorimotor synchronization have been documented, namely tapping synchronization from childhood through adulthood, and corporeal synchronization only in early childhood and adulthood. Based on the available literature so far, we cannot yet answer the questions: at what age can children reliably synchronize their full body movement to the beat, and at what age do their synchronization abilities reach the level seen in typical adults?

The aim of this study was to observe basic bodily rhythmic synchronization abilities during middle childhood (ages 6 through 11 years) in comparison with performance in adults. To help fill the gap between reports of mainly undeveloped bodily synchronization in young children, and quite reliable bodily synchronization in adults, we aimed to provide an indication of this skill, as measured by period-locking and phase-locking to the beat of auditory metronomic and rhythmic stimuli at two different tempi. We did this following the methodology of prior work^40^ using a wireless motion capture device to record body movement data, from which we analyzed the proportion of energy at the musical beat period (and related frequencies), and the degree of phase-locking to those frequencies. This experiment is meant to serve as an initial step in understanding full-body rhythmic synchronization in middle childhood.

## Results

### Period-locking

**Figure 1:**
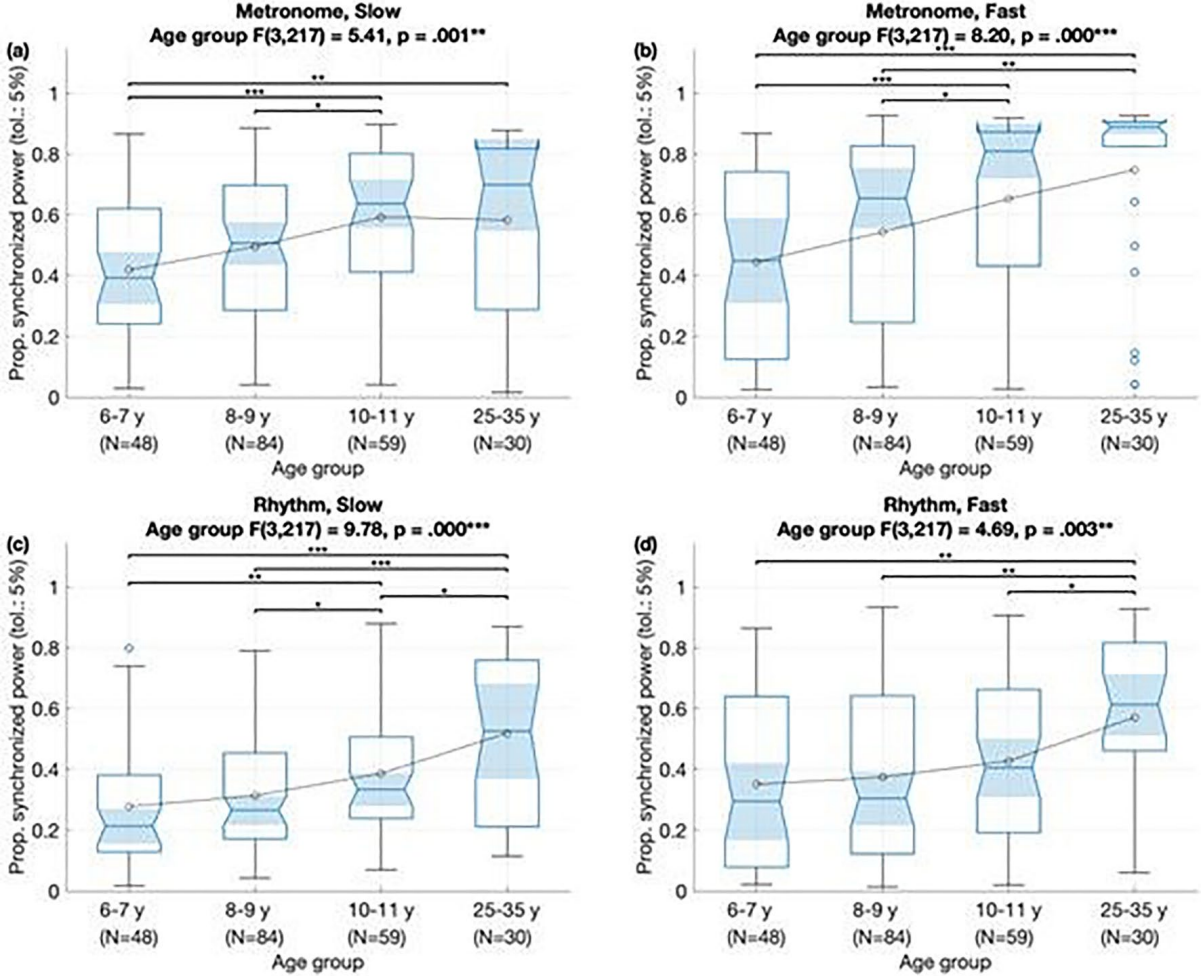
Boxplots show results of analyses for period-locking (as measured by proportion of synchronized power) by age group for each stimulus: (**a**) slow metronome, (**b**) fast metronome, (**c**) slow rhythm, and (**d**) fast rhythm.

We analyzed whether the adherence of the body movements to the musical beat level and metrically related frequencies could be explained by demographic and musical factors. To this aim, we conducted type III ANOVA marginal tests for a linear mixed-effects model with period-locking as a dependent variable and with age group, gender, stimulus type, stimulus tempo and their two-way interactions as predictors plus participant as a random intercept. We found significant main effects of age group, stimulus type, and tempo on period-locking performance (see Fig. 1). Period-locking improved significantly with age group, *F*(3861) = 12.77, *p* < 0.001. In addition, period-locking performance was better for the metronome than for the rhythm stimulus, *F*(1861) = 105.37, *p* < 0.001, and better for faster than slower tempo, *F*(1,861) = 18.09, *p* < 0.001. There were no significant interactions between the predictors. Results followed a similar trend after removing the adult group (25–35 y) from the sample to ensure it was not biasing the overall results and adding parent education as predictor, yielding *F*(2727) = 7.82, *p* < 0.001 for age, *F*(1727) = 50.61, *p* < 0.001 for stimulus type, and *F*(1727) = 7.12, *p* < 0.01 for stimulus tempo. We also found a significant interaction between age and stimulus type, *F*(2727) = 3.08, *p* < 0.05 for stimulus tempo: compared against 6–7 year olds, period locking of older children was higher for metronome stimuli than for rhythmic stimuli.

## Phase-locking

**Figure 2:**
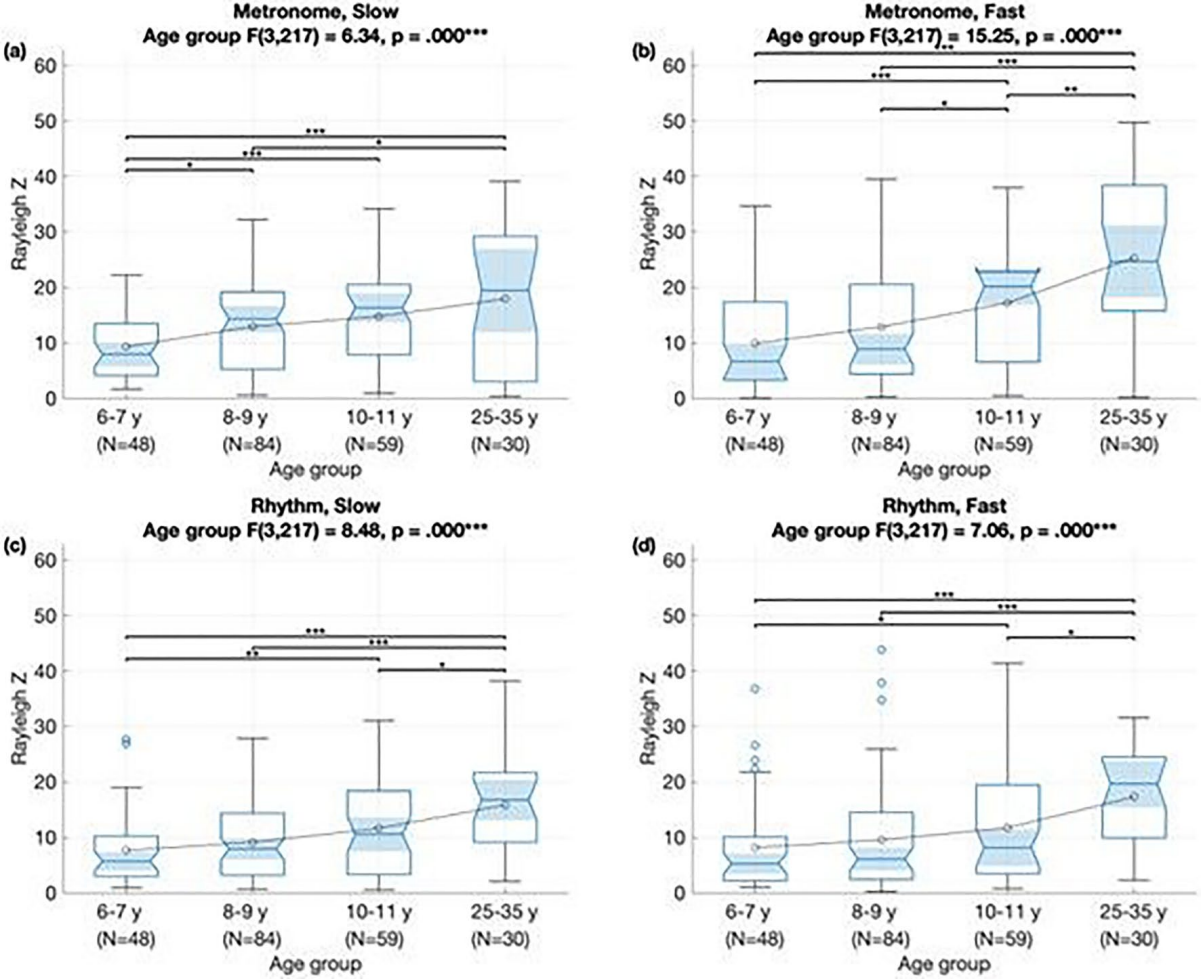
Boxplots show results of analyses for phase-locking (as measured by Rayleigh Z statistic) by age group for each stimulus: (**a**) slow metronome, (**b**) fast metronome, (**c**) slow rhythm, and (**d**) fast rhythm.

Next, we investigated the effect of demographic and musical variables upon the degree of period-locking and constant phase of the rhythmic movements. Again we ran type III ANOVA marginal tests for a linear mixed-effects model with phase-locking as a dependent variable and with age group, gender, stimulus type, stimulus tempo and their two-way interactions as predictors plus participant as a random intercept. Results showed a significant effect of age, *F*(1861) = 16.48, *p* < 0.001, and of stimulus type, with phase-locking significantly better for metronome than for rhythm stimuli, *F*(1861) = 50.98, *p* < 0.001 (Fig. 2). The results also showed a significant interaction between age group and tempo, *F*(3861) = 2.62, *p* < 0.049. Specifically, compared against 6–7 year-olds, phase-locking scores in 8–9 year olds were significantly higher for slower stimuli, whereas phase-locking scores in 10–11 year olds were significantly higher for faster stimuli. A similar pattern of results was obtained after removing the adult group and adding parent education as predictor, i.e. *F*(2727) = 7.9, *p* < 0.001 for age and *F*(1727) = 12.19, *p* < 0.001 for stimulus type.

## Phase peak in movement versus auditory beat

**Figure 3:**
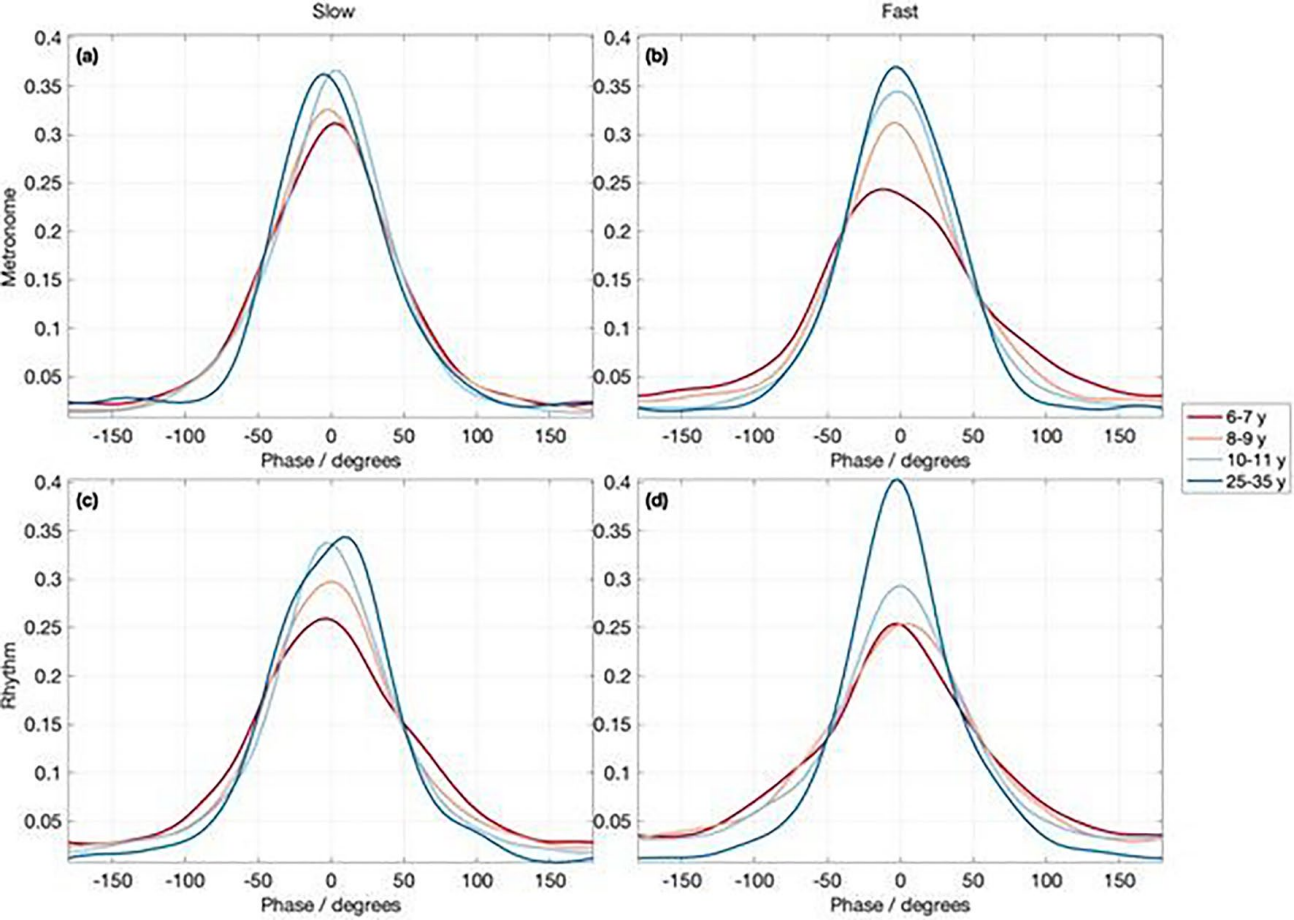
Results of the Von Mises distributions indicate the degree of peak phase shift, calculated for individual participants’ optimal beat levels, by age group for each stimulus: (**a**) slow metronome, (**b**) fast metronome, (**c**) slow rhythm, and (**d**) fast rhythm.

In order to examine where the participants’ peak accelerations (that is, the execution of the “beat” onset in their bouncing movement) occurred with respect to the beat location in the auditory stimulus, we calculated mean averaged von Mises distributions across participants. A participant can yield an identical von Mises distribution regardless of whether they are in-phase, anti-phase or quarter-phase aligned to the stimulus. The phase of the maximum of the von Mises distribution tells us how phase-shifted the individual tends to be with respect to the mean angle of the phase difference between the accelerometer and the music. Thus, these distributions show the degree of peak phase shift, for individual participants’ optimal beat levels (Fig. 3).

## Towards a baseline measure for children’s synchronization by age: preliminary observation

The present data on rhythmic synchronization in children are extremely varied, and the component skills of period- and phase-locking seem to develop slowly over many years. Importantly, the stimuli for this study were chosen for the purpose of initial comparison with available adult data on full body synchronization to rhythm versus metronome stimuli, and thus do not represent optimal performance for children. Nevertheless, out of the stimuli used and the components measured in the present study, we wanted to observe which stimulus, if any, was effective in reliably separating participants by age on synchronization ability. To this aim we used a two-sample Kolmogorov–Smirnov test, comparing the mean von Mises distributions (from -π to π) from all pairs of age sub-groups (Table 1). The results showed that for phase-locking with the fast metronome, the null hypothesis was rejected for all age comparisons, meaning that the curves are different between age groups for performance on this measure and stimulus. While this test showed that all 4 conditions succeeded on differentiating at least four out of the six age groups, and the size of the effects are not necessarily the highest for the fast metronome in all cases, this preliminary observation suggests that measuring phase-locking with a metronome at 124 bpm (2.07 Hz) is one fairly reliable metric for tracking change in the ability to synchronize to an auditory stimulus during childhood. Future studies utilizing stimuli within the range of spontaneous motor tempo for children will be helpful in refining the conditions that best characterize children’s synchronization ability.

**Table 1:** Results of two-sample Kolmogorov–Smirnov test for phase-locking.

Stimulus	Comparison	RejectH	*p*	Ks2stat
Metronome: Slow	6–7 and 8–9 years	True	0.031377	0.2
	6–7 and 10–11 years	True	0.00017421	0.3
	6–7 and 25–35 years	False	0.13998	0.16
	8–9 and 10–11 years	True	0.020495	0.21
	8–9 and 25–35 years	False	0.069092	0.18
	10–11 and 25-35 years	True	0.00032154	0.29
Metronome: Fast	6–7 and 8–9 years	True	0.0010291	0.27
	6–7 and 10–11 years	True	2.7524e-07	0.39
	6–7 and 25–35 years	True	1.0553e-11	0.5
	8–9 and 10–11 years	True	4.8052e-05	0.32
	8–9 and 25–35 years	True	5.6969e-10	0.46
	10–11 and 25–35 years	True	0.0010291	0.27
Rhythm: Slow	6–7 and 8–9 years	False	0.099376	0.17
	6–7 and 10–11 years	True	0.0017847	0.26
	6–7 and 25–35 years	True	1.2116e-07	0.4
	8–9 and 10–11 years	False	0.13998	0.16
	8–9 and 25–35 years	True	5.9565e-06	0.35
	10–11 and 25–35 years	True	0.00058125	0.28
Rhythm: Fast	6–7 and 8–9 years	False	0.19304	0.15
	6–7 and 10–11 years	True	0.0017847	0.26
	6–7 and 25–35 years	True	2.1683e-10	0.47
	8–9 and 10–11 years	False	0.44313	0.12
	8–9 and 25–35 years	True	9.122e-09	0.43
	10–11 and 25–35 years	True	1.466e-09	0.45

Results of the two-sample Kolmogorov–Smirnov test indicate that performance in phase-locking with the fast (124 bpm) metronome is a reliable metric for tracking children’s rhythmic synchronization performance with age.

## Discussion

Period- and phase-locking in full-body synchronization to metronome and rhythm stimuli improved significantly between the ages of 6 and 11 years. They did not reach adult levels however, indicating that as fundamental as these entrainment processes seem to be, they nevertheless develop gradually throughout childhood, and potentially through adolescence. As we did not measure older children and adolescents (ages 12–24) here, future studies will be needed to measure improvement in performance during that period of development.

Both period- and phase-locking were better for the simple, isochronous metronome stimuli than for rhythm stimuli across age groups, which appears to be consistent with some previous results of full-body synchronization to a metronome versus more complex drum rhythm stimuli^51^. In the present study, period-locking was also better for the fast than for the slow tempo, results which differ from prior results with metronome and drum rhythm stimuli in adults showing no significant difference in tempo^40^. This is likely attributed in part to differences in spontaneous motor tempo or optimal tempo range between children and adults^39,52^, as well as the beat salience or complexity of the drum rhythm patterns between studies. As noted in the introduction, the present study was intended to provide a first step in observing how performance in children compares to adults with previously used stimuli. Follow up studies will need to examine period- and phase-locking performance on a more optimal or preferred range of tempo in middle children.

The present phase-locking data showed significant improvement with age, as well as significantly better performance for metronome than for rhythm stimuli. However the results also indicated an interaction between age group and tempo among the children, which we explain here. While the individual contrasts show an overall pattern of improvement with age, two notable jumps in phase-locking scores stand out that account for the interaction, especially with the metronome stimulus. The first jump in improvement is between 6–7 years and 8–9 years for the slow tempo (with non-significant improvement between 8–9 years and 10–11 years), and the second jump in improvement occurs between 6–7 years and 10–11 years for the fast tempo (with non-significant improvement between 6–7 years and 8–9 years). These results suggest that improvement in phase-locking may be less linear than that of period-locking, instead showing somewhat discrete jumps in ability at a slightly younger age for this slow tempo, and then again a couple of years later for this fast tempo, in particular with the metronome stimulus. A significant question that remains is: do phase locking abilities emerge around the age of 6–7 years, or are these skills pre-existing? In further exploration, we found that over 75% of all the Rayleigh tests conducted for the 6–7 year age group yielded statistically significant results at *p* < 0.05. This implies that phase locking skills are generally present by the age of 6 and continue to develop with age. Future studies exploring a wider tempo range, and individual tempi selected for optimal performance before and throughout this period of pre-adolescent development, will help further elucidate the developmental trajectory of temporal synchronization skills.

Children’s behavioral data are notoriously variable, and data on musical abilities in general—and rhythmic synchronization in particular—are no exception. From the present data, we found that one metric was notably reliable in terms of tracking change in sensorimotor synchronization with an auditory stimulus across age groups: that was phase-locking with a metronome at 124 bpm (2.07 Hz). We suggest that future studies examining rhythmic synchronization in children might use this metric for tracking age-related changes, keeping in mind however that it may not be an optimal measure of musical ability. That is, synchronization to more complex (not purely isochronous) musical rhythm patterns, or at different tempi, may ultimately be more relevant to the perceptual and cognitive skills associated with music practice.

In addition to the limitation of the stimuli in the present study is the limitation of the type of movement measured: that is, in an effort to compare children’s performance with that of adults, we asked them to perform the synchronization task in the same manner as that used in prior studies with adults. While there is evidence of the important role of vertical head and body movement in entrainment^13,53,54^, there is also evidence of much variation in children’s and adults’ preferred or potential movement patterns (such as dancing, drumming, tapping or head bobbing), as well as preferred musical stimuli^18,30,31,37^—all of which can affect their precision in timing with respect to a musical beat. It is also important to note that while numerous factors can influence individuals’ preferred or optimal movement patterns for sensorimotor synchronization, it can not be taken for granted that various types of movement (e.g., discrete versus continuous, fine versus gross motor, limb versus head, or single versus multiple simultaneous moving parts) are equivalent indices of rhythmic entrainment ability or its underlying mechanisms^53,55–57^. Future studies will need to address how rhythmic timing and synchronization performance of children and adults varies based on type of corporeal movement and musical stimulus.

In sum, full-body rhythmic synchronization ability is undergoing development but is not fully developed before age 12, as the present comparisons show better performance for period- and phase-locking in adults than in all age groups during middle childhood. We therefore have reason to measure improvement between 12 and 24 years of age, in order to determine when performance typically becomes adult-like. Of particular interest might be examining at what developmental stage period- and phase-locking abilities each reach adult levels. We predict that data collected during adolescence will show that period-locking reaches mature levels at a younger age than phase-locking. Period-locking can be considered a more basic rhythmic synchronization skill in that it requires only extracting the beat period (frequency) without regard for placement of the onset of the movement (phase alignment). In contrast, phase-locking is a compound skill that requires both the accurate period frequency and alignment of the movement beat onset with respect to the auditory beat onset. That is, period-locking does not require phase-locking with the external beat, but phase-locking requires period-locking. As musical rhythmic entrainment ability develops, we expect to see continued improvement in both, with phase-locking ability building upon and reaching maturity after the elemental skill of period-locking.

Finally, we offer a few words about the potential for children to show precocious musical behavior. While the study of skills like rhythmic synchronization in groups of ‘typical’ children—that is, individuals who are not selected for extensive musical exposure or ability—is useful for understanding the time course of development of those skills in general during childhood, these studies do not reveal what is possible in children at those ages. An online search for young talented musicians, even toddlers, reveals examples of seemingly advanced rhythmic skills at young ages, whether keeping the beat, anticipating the break, or performing advanced rhythms ^36,58–60^. In addition, children in cultures and communities that frequently incorporate rhythm through song, dance, drumming and other activities into daily life can show abilities that are much more advanced than we see in results such as the present study, and they are not necessarily represented in the population samples of lab studies such as this one.

## Method

### Participants

#### Child sample

Two hundred one children between the ages of 6 and 11 years (M_age_ = 8.50, SD = 1.47, 50% female) were recruited from a public school. The participants were recruited as part of a more extensive project whose main objective is to explore the relation between executive functions and the ability to synchronize full-body motion to rhythm patterns in deaf and hearing children (project reference: PID2019-111454RBI00/AEI/10.13039/501,100,011,033).

Of the 201 children recruited, 11 children were excluded due to the following reasons: (1) presented learning and/ or neuropsychological disabilities (attention deficit hyperactivity disorder, autism spectrum disorder) and (2) did not want to complete all the tasks used in this study. The final sample of children consisted of 190 children considered to be typically developing between 6 and 11 years (M age = 8.65, SD = 1.50, 50.5% girls). They were divided into three age groups (6–7 years, 8–9 years, and 10–11 years), coinciding with their grade in primary school.

Information regarding the age, gender and educational level of the children’s parents is in Table 2. The level of education of the parents of children were indicated as one of four levels: low (6 to 8 years and/ or ESO), medium (High school and Higher grade), high (University studies) and, unknown (parents who did not indicate their education level). When both parents reported their educational level, in order to obtain a single score, the highest educational level of both of them was taken into account in this study. The chi-square test revealed differences between the 3 age groups related to the educational level of the parents, *X*^*2*^ (6) = 14.503, *p* = 0.024, but not according to gender, *X*^*2*^ (2) = 3.571, *p* = 0.168.

**Table 2:** Sociodemographic data of participants.

Group of ages (years)	6–7 (n = 48)	8–9 (n = 83)	10–11 (n = 59)	Adults (n = 30)
Age (Mean, Standard Deviation)	6.6(.5)	8.6(.5)	10.4(.5)	28.3(3)
Gender *f* (%)				
Male	23(47.9)	47(56.6)	24(40.7)	14(46.7)
Female	25(52.1)	36(43.4)	35(59.3)	16(53.3)
*Parent Education f (%)*				
Basic	7(14.6)	17(20.5)	23(39)	
Medium	14(29.2)	34(41)	13(22)	
High	25(52.1)	29(34.9)	20(33.9)	
Unknown	2(4.2)	3(3.6)	3(5.1)	

We obtained information from parents regarding any kind of music training of the children. However, of the 190 children, only 27 had experienced music lessons, and of those, 21 were girls. Since we had so few data points on music training at this young age, we decided not to include it as a factor in our analyses.

## Adult sample

Thirty adult volunteers between 25 and 35 years of age (M_age_ = 28.33, SD = 3.04, 53.33% female) were recruited from social media advertisements. Participants had no known neurological or psychiatric conditions and were considered representative of normo-typical development. Table 2 presents information regarding age and gender of this group. As we only evaluated college students, we did not make comparisons between groups regarding level of education. Exploratory analyses showed no significant differences for gender distribution between child and adult groups, *X*^*2*^(3) = 3.654, *p* = 0.301.

## Procedure

Approval was obtained from the Human Research Bioethics Committee of the University of Almería (approval number: Ref:UALBIO2019/020). The research was performed in accordance with the applicable guidelines and regulations for testing human subjects and in accordance with the Declaration of Helsinki. An informed consent was obtained from every participant, parent or guardian before participating in the study.

The task was administered to children individually in a separate and quiet room of the school during the school day. The mean time to complete all the tasks was approximately 7 min with breaks in case the participant began to show signs of fatigue. For the adult group, the experiment was conducted in the Basic Psychology Laboratory of the Department of Psychology at the University of Almería. The volunteers gave their written informed consent to participate, and for all participants they received the same instructions as children.

The task used to measure rhythmic synchronization was similar to that used by Phillips-Silver and colleagues^51^. Participants were instructed to bounce in place by bending their knees to the beat of two types of auditory stimuli each at two different tempi: an isochronous metronome, and a simple rhythm pattern in drum timbres. The fast and slow tempi were chosen for this original study because they are distinguishable but fall around the common preferred tempo of adult human motion and dance^52^. Tempi and beats of audio files were obtained using MIR Toolbox^61^. The bouncing movement was captured with an accelerometer in the Nintendo Wii remote control, which was attached to the participant’s waist. This device measured three dimensional acceleration of body movement (bouncing) with a temporal resolution of 100 frames per second (10 ms).

Participants first practiced the task following the example of the experimenter during 10 s of an isochronous metronome. Once the evaluator observed that the participant understood the task, they were presented with the four stimuli, for four trials. In between stimuli participants were asked if they needed to rest in case of fatigue. The same instructions were presented for children and adult samples.

Participants bounced to the beat of each type stimulus at the faster and slower tempo. The auditory metronome stimulus at the slow tempo was 99 beats per minute (BPM), corresponding to 1.65 Hz; this stimulus had a total of 68 beats and a duration of 41 s. The auditory metronome at the fast tempo was 124 BPM, corresponding to 2.07 Hz; this stimulus had a total of 72 beats and a duration of 33 s. The drum rhythm stimulus at the slow tempo was 98 BPM, corresponding to 1.63 Hz; this stimulus had a total of 68 beats and a duration of 44 s. The drum rhythm at the fast tempo was 123 BPM, corresponding to 2.05 Hz; this stimulus had a total of 72 beats and a duration of 35 s.

## Feature extraction from Wii motion capture data

Three dimensional acceleration data was extracted from the captured bouncing motion using MoCap Toolbox^61^. Prior to the computation of period- and phase-locking measures, data was trimmed by 5 s from the length of shortest recording (23 s), resulting in a total duration of 18 s. Principal component scores from the first principal component were computed so as to focus on the movement direction that maximized the variance in the data. This was performed to minimize effects of possible differences in the orientation of the Wiimote between participants.

## Measures of rhythmic synchronization

Period-locking refers to the degree of adherence of the period of rhythmic movements to the musical beat level and metrically related frequencies. This is equivalent to the measure of BPM or Hertz. Following previous work, a Fourier analysis was performed to measure the overall proportion of power in the Fourier spectra (within the range of 0 to 5 Hz) at the musical beat level and associated frequencies (half and double the musical beat level), with a 5% tolerance window for error [see^40,51^]. The chosen tolerance window provided the clearest distinction between the age groups.

Phase-locking describes the extent to which the rhythmic movements are period-locked and maintain a phase constant over time. By performing a Hilbert transform on the movement data, the measure calculates constancy of the difference between the instantaneous phase of a continuous wave derived from the musical beat level (or a metrically related frequency) and the instantaneous phase of the rhythmic movements^40,51^. Rayleigh’s Z test for circular uniformity is computed from phase differences at the musical beat level and metrically associated frequencies, from which a maximum Rayleigh’s Z-value across metric levels is used as a measure of phase-locking.

Von Mises distributions can be used to inspect both the degree of phase-locking and whether the bounces tend to be anticipated or delayed with respect to the average phase difference between the movement and the music. To compute them, phase differences are further expressed as directional vectors and centered based on their mean angle. For the metric level that maximized the Rayleigh’s Z of each participant, and for each time point of each recording, a von Mises distribution (κ = 15) over the interval [–π, π] is obtained and scaled to [0,1]. Next, for each participant and stimulus, the distributions are mean-averaged across time points. Finally, the distributions are mean-averaged across participants separately for each stimulus and age group.

## Data Availability

The datasets generated during and/or analyzed during the current study are available from the corresponding author on reasonable request.
